# The plight of prestige: The subjective experiences of women managing chronic physical illnesses and their emotional well-being in the context of the prestige hierarchy

**DOI:** 10.1177/13591053251329675

**Published:** 2025-03-31

**Authors:** Carly Hook, Zoe Palfreyman, Ben Gibson, Nadzeya Svirydzenka

**Affiliations:** De Montfort University, UK

**Keywords:** women’s health, chronic illness, emotional well-being, qualitative methods, interpretative phenomenological analysis (IPA), prestige hierarchy

## Abstract

Chronic illnesses, such as diabetes and epilepsy, impact millions globally. Despite the burden of chronic illnesses, a medical hierarchy exists, with many illnesses undervalued in society, hence allocated minimal research funding. This bias disproportionately affects health outcomes for women. This research provides a novel exploration into the lives of women with chronic illnesses of varying levels of prestige, examining commonalities and variations among their illness experience, and the coping strategies they employ to manage their emotional well-being. Six semi-structured interviews were conducted and analysed using Interpretative Phenomenological Analysis. Two superordinate themes were developed: “A fractured reality” and “A restrained reality.” Commonalities across the narrative were manifested in structural inequalities and coping strategies, however, illnesses lower on the prestige hierarchy were evident with an existential conflict with the illness identity. This research demonstrates the structural discrimination of the gender construct and the disparities experienced by women with conditions of lower prestige.

## Introduction

Within society, chronic illness is often perceived through a reductionist lens, with illness without a physical cause as fictitious, in accord with the biomedical model of medicine (Burke, 2019). This magnifies the prejudicial view that such illnesses are psychological (Tesio and Buzzoni, 2020), increasing the risk of patients experiencing disempowerment within societal and structural systems. Life with chronic physical illness (CPI) brings intrinsic and extrinsic challenges on a multidimensional scale (Knowles et al., 2020). Such challenges, including the burden of symptoms or stigma can reduce emotional well-being (EWB) (Hudson and Moss-Morris, 2019). Conversely, low EWB can exacerbate symptoms in many CPIs, such as pain intensity in Arthritis (Ziarko et al., 2019), an increased likelihood of further cardiac events in heart disease (Pimple et al., 2019), and a compromised prognosis of those with cancers and mental illness (Jayatilleke et al., 2017; Petty and Lester, 2014). Healthcare practice for individuals with all CPIs should incorporate a biopsychosocial approach, accounting for EWB and health-related quality of life, yet many healthcare professionals (HCP) continue to strictly adhere to the biomedical model of disease (Kusnanto et al., 2018; Wierenga et al., 2017). CPIs are socially constructed and often grouped together and yet despite the substantial growth of understanding within medicine and broader society, not all CPIs receive equal recognition, and subsequently many are under-valued. HCP perceptions of disease prestige are ingrained, and low-prestige CPIs are consistently down-prioritised, receiving less funding for research, exacerbating health disparities (Hindhede and Larsen, 2020).

According to projections from the United Nations, 1.5 billion people will be over the age of 65 by 2050, with women typically outliving men (United Nations, 2022), hence women have an increased likelihood of living with a life-impacting CPI (Benziger et al., 2016). Health research has historically focused on men’s health and anatomy, leaving women’s physiology, needs, and symptoms underrepresented. The trend continues, with women more likely to report the dismissal of their illness experience (Braksmajer, 2017; Thompson and Blake, 2021). Gender disparities are well documented across healthcare settings, contributing to psychological and socio-cultural inequalities, permeating through interpersonal interactions and structural systems (Jaworska and Ryan, 2018). Despite an increasing awareness of the gender bias within health, economic distribution of money allocated for health research continues to disadvantage women (Ballreich et al., 2021; Mirin, 2021). Research evidences a perceived medical hierarchy of disease amongst HCP, with diagnosable and treatable conditions of high prestige, such as heart disease, whereas those low on the scale, such as Fibromyalgia, are typically harder to diagnose and have minimal treatment options (Hindhede and Larsen, 2020). In accord, high prestige illnesses are allocated more economic funding, with decreased allocated funding for those lower on the scale. Subsequently, a vast number of life-impacting illnesses and syndromes, disproportionately affecting women, place considerable burden on patients, healthcare systems, and the wider economy, and yet receive minimal economic funding (Album et al., 2017). This is misguided, as if more funding was allocated to illnesses low on the medical hierarchy, it would reduce the burden on healthcare systems, reducing the cost to the patient and economy. A reductionist discourse continues amongst HCP, society, and policymakers, ergo certain illnesses will continue to be down-prioritised, with less research and understanding until this discourse is addressed (Tesio and Buzzoni, 2020).

With the transition to patient-centred care, individuals with CPIs are assuming a more proactive role, including medication adherence and lifestyle adaptations, providing an opportunity to regain a sense of control over their outcomes (Timmermans et al., 2021). This affords patients the right to incorporate their own values and belief systems throughout the illness trajectory. Yet with research indicating the down-prioritisation of certain CPIs, the likelihood of holding positive illness representations to facilitate self-management behaviours is reduced. Informed by cognitive, behavioural, and perceptual responses to health threats, self-management behaviours are underpinned by illness perceptions (Leventhal et al., 2016). Such perceptions inform action plans for self-management behaviours and coping strategies which are reappraised and adapted throughout the illness trajectory (Hagger and Orbell, 2003). A complex interaction of intrinsic and extrinsic barriers can hinder engagement in such behaviors. Intrinsically, individuals must have self-efficacy, locus of control, and health literacy to actively undertake responsibility for their care (Van de Velde et al., 2019). Extrinsically, individuals must be fully informed about their CPI and the importance of self-management behaviors (Cheen et al., 2019). If individuals are not fully informed, there are minimal resources available, or receive suboptimal care, illness representations could be negatively impacted (Homma et al., 2018). Subsequently, patients may resort to maladaptive coping strategies leading to symptom exacerbation, psychological distress, and reduced health-related quality of life (Knowles et al., 2020).

### Present study

An ingrained bias in women’s health remains, with considerable evidence highlighting the inequality and disparities surrounding the quality, availability, and accessibility of women’s healthcare services (Mauvais-Jarvis et al., 2020; Thompson and Blake, 2021). Inequalities have been highlighted for individuals with CPIs with no biological cause, which are low on the prestige hierarchy, and receive minimal economic funding (Ballreich et al., 2021; Burke, 2019). Such individuals can experience CPI burden for many years before receiving a diagnosis and subsequently, a continued disempowerment within society (Braksmajer, 2017). Engaging women with a variety of CPIs both with and without a well understood biological aetiology, to identify interpersonal and socio-economic commonalities amongst their experiences would further the topic area. A subjective principle, EWB can be impacted through the episodic nature of the CPI, the intrinsic and extrinsic challenges, or with the vicissitudes of the illness trajectory. As such, providing a psychological insight of how women make sense of these challenges to manage their EWB would advance on the literature. This study aimed to investigate women’s lived experiences of various CPIs, of high and low prestige, to identify commonalities and variations across different diagnoses, and the influence on their emotional well-being.

The study questions are as follows:

What are the impacts of CPI of varying perceived prestige on the lives of women?

How do women establish and maintain coping strategies to manage their lives?

How do women feel their CPI affects their EWB?

## Method

### Design

Within health psychology, a significant importance is given to the patient’s perceptions of their health, and the interpretations and meanings they place on their bodily experience (Newman et al., 2008). Subsequently, semi-structured, one-to-one interviews were selected as the most appropriate approach for data collection, with an exploration into the lived experiences of women living with a variety of CPIs. The semi-structured nature provided a guide to the conversation, enabling participants to lead the narrative, highlighting and elaborating on the experiences of significance to themselves, whilst engaging in experiential and existential self-reflection (Nizza et al., 2021).

### Participants and recruitment

Participant recruitment was conducted through a UK university’s student research platform SONA, with participants receiving course credits for taking part. Interested participants who fit inclusion criteria could sign up to the study online and contact the researcher at the email address provided. Inclusion criteria required participants to be aged 18 or over, female, and diagnosed with a CPI. Six participants self-selected as eligible for the study, with an age range of 19–44 (*M* = 28.5, SD = 9.53) each with a CPI (see Table 1). With the emphasis of IPA on the in-depth analysis of each transcript the sample size is appropriate, in keeping with prior research (Smith and Osborn, 2007). Participants provided electronic informed consent prior to being interviewed.

**Table 1.** Participant characteristics

Pseudonym	Age	CPI
Amelia	20	Rheumatoid arthritis
Josie	39	Fibromyalgia
Anne	19	Ehlers-Danlos Syndrome and Fibromyalgia
Charlotte	25	Solitary Rectal Ulcer Syndrome
Jessica	24	Epilepsy
Sophie	44	Diabetes Mellitus

### Data collection

Ethical approval was obtained from De Montfort University. Once participants registered their interest in the study by emailing the researcher, they were emailed an information sheet and consent form to complete before being interviewed. Participants were informed of their right to withdraw on the information sheet and consent form and were advised that withdrawal 3 days (72 hours) after the interview would not be possible. Whilst a short turnaround period, this ensured that data analysis could begin within the time restraints for the research project. Interviews were conducted via Microsoft Teams, and before recording, participants were reminded of their rights to withdraw and could have any questions answered by the researcher. Interviews were conducted with and without their camera switched on, as to their preference, and audio-recorded on an encrypted Dictaphone. A semi-structured interview schedule was developed, with 12 open-ended questions. To avoid preconceived aims and beliefs of the author, questions were broadly informed by prior research, including the domains of the Common-Sense Model (Leventhal et al., 2016). The schedule encompassed psychosocial questions within the biological context of the CPI, with examples such as:

1. Can you tell me about the influence of your illness on your life?
2. Thinking about your illness, from the beginning of your symptoms to today, have you ever been judged negatively?
3. Can you describe how your illness influences you emotionally? How do you manage your well-being?

Following their interview, participants were emailed a debrief sheet, containing contact details for the research team, and details of support available at Mind.Org, and the Samaritans.

### Data analysis

An Interpretative Phenomenological Analysis has been employed as the most appropriate method for an exploration into the subjective experiences of women living with a variety of CPIs. With experiences that differ between two people, the process of IPA enables an interpretation of how participants understand their condition and how it impacts their lives (Smith et al., 1999). Commonly used in Health Psychology, the idiographic nature of IPA provides an understanding of the lived experiences of each participant living with a CPI, the impact it has on EWB, and their reflections of the significance of the illness (Smith and Osborn, 2007). All interviews were transcribed verbatim, and each participant was provided with a pseudonym to ensure anonymity (Table 1). The first transcript was analyzed using a free text analysis, before a full immersion within the narrative, drawing on how the participant had made sense of their illness experience. Notes were made in the margin, before being transferred to a table and grouped into developing themes. This process was then repeated across each case, before transcripts were examined collectively, highlighting any convergence or divergence across the whole set of interviews, before identifying any patterns in how participants reflected upon shared experiences (Smith et al., 1999). As the analysis continued, superordinate and subordinate themes were developed and illustrating the part to the whole aspect of IPA, unfolded into one narrative (Nizza et al., 2021).

## Results

Following the engagement with the narrative, two key superordinate themes were developed to reflect the experiences of participants (Table 2): (1) “A fractured reality” and (2) “A restrained reality”. Subthemes for the former illuminate the struggle to have their illness experience legitimized, and the process of adjusting to a new disabled identity. The latter highlighted interpersonal to structural level disparities, with a fear of the perceived stigma, and a sense of disconnection from their worlds.

**Table 2.** Theme descriptions

Superordinate Theme	A fractured reality	A restrained reality
Subtheme 1	Fighting for low-prestige legitimization	Fear of injustice
Subtheme 2	Fragmented identity	Bound by society
Subtheme 3	Striving for normality	Holding onto hope

### Superordinate theme 1—A Fractured Reality

#### Subtheme 1—Fighting for low-prestige legitimization

The onset of a CPI has been conceptualized as a process of self-transformation, through which one adapts to the biopsychosocial demands of the illness. This can be a time of turmoil and disruption as individuals navigate their way through the embodiment of their new disabled selves, with the legitimization of a diagnosis or pushing to be heard. With high-prestige CPIs, Sophie, with Diabetes, and Jessica, with Epilepsy, received a timely diagnosis and were provided with prescription medications. However, the remaining participants live with CPIs of lower prestige, which could not be easily diagnosed or treated. Their diagnostic trajectories evoked feelings of anger and frustration at the healthcare system highlighting the down-prioritization of low- prestige conditions. Participants were all in their adolescent years at the start of their symptoms, ergo the invalidation was potentially magnified due to the early onset of their conditions. Amelia highlights the imbalance of power within the doctor-patient encounter:

The amount of times you’d go… but it was just, oh no, you’re fine… or that you’re just making it up… How many times do you have to go to the GP… and be like… I’d appreciate it if you could do some X-rays… because I’m telling you it’s not normal (Amelia, 20)

This experience was tangible across the narrative, as participants spoke of the challenges they had to overcome to reach diagnosis. Despite the availability of clinical testing for the CPIs experienced by Amelia and Charlotte, one could argue that there may have been a reluctance in HCP in sending the women for these tests, potentially indicative of economic restrictions. This delay, however, caused an exacerbation of their symptom and illness experience, which could have been avoided, as Charlotte, with Solitary Rectal Ulcer Syndrome reflects:

I was back and forth… my GP… referrals to paediatric departments… back and forth, until I finally had a colonoscopy… they said ‘oh, there’s something there, that’s what it is… so basically… Finally, someone had found a cause, or the result of the problem… (but) it was just, ‘here are all these laxatives’ (Charlotte, 25)

Josie’s transcript symbolised the down-prioritisation of Fibromyalgia, with the lack of understanding and awareness of the condition amongst her HCP and her family and is still observed within Fibromyalgia research today. Explained away as growing pains, this discourse negates her illness experience, leaving her bewildered, and isolated from her peers:

No-one knew anything about it… All everyone used to say is, your body is changing… I started putting on weight… everyone kept saying… you should be out and about… But they just didn’t know (Josie, 39)

Whether being dismissed was due to their age, articulation of symptoms or diagnostic uncertainty remains uncertain, the influence on their self-concept and negative perceptions of HCP was evident with all. For Anne, with Ehlers-Danlos Syndrome and Fibromyalgia, the years waiting for diagnosis forced them with no option but private healthcare. Whilst confirmation of her diagnoses was initially perceived as a joyous occasion, finally achieving the validation, reality hitting became a stark contrast:

Decided to celebrate it. Mum got me a cake with candles, like it was my birthday. We did happy diagnosis day… I’d decided that I was going to think of this positively and celebrate it… but then you’re left to settle… and that’s the hard bit (Anne, 19)

#### Subtheme 2: Fragmented Identity

Following their diagnosis, the reality of life with a CPI interrupted their process of self-transformation, as they each struggled with acceptance of their new disabled identity. Despite the variety of high and low-prestige illnesses, a sense of negotiation with their new self-concept was apparent for each participant. Sophie’s concerns about asking for support from her university highlights an internal struggle, perceiving asking for help as a weakness, regardless of how her illness may be affecting her:

I hate… that’s a strong word… but I really do hate taking advantage… I’m sitting and thinking, I have to write an essay… but I just can’t… then I get stressed. So, I ask for an extension… I think how can I prove that? What are they going to think of me? (Sophie, 44)

The internal struggle of the invisible illness experience and trust that others in society will accept her at face value was mirrored in Jessica’s narrative, strongly believing that as she does not look chronically ill, she should be healthy. Her perceptions of all people viewing her negatively suggest that she is emotionally bypassing her true feelings, and instead draws on her anger to shift blame onto others:

When I’m on the bus… I get a lot of people staring at me, thinking… erm… Why has she got a bus pass… You haven’t got a disability… Yes, I have, just ‘cos it’s invisible doesn’t mean it’s not real (Jessica, 24)

For Anne, despite her clinical diagnoses of Ehlers-Danlos Syndrome and Fibromyalgia, an internal struggle exists with her perceptions that she is not disabled enough. This perception contradicts her illness experience, concerned about how she feels she should meet ‘societal expectation’ of disabled likely leads to her not fully accepting the illness identity, and instead leads to self-dismissal. As she highlights:

Severe imposter syndrome… severe, severe, imposter syndrome, erm, despite living with it my whole entire life… felt like I wasn’t disabled enough because all of my joints wouldn’t properly dislocate. That I wasn’t disabled enough to classify as EDS… (Anne, 19)

For Charlotte, the lack of societal understanding and awareness of her Solitary Rectal Ulcer Syndrome has left her feeling detached from those with more readily understood illnesses. Her reflections illustrate her conflicting perceptions of her own identity, not feeling she truly belongs:

It’s not like I’ve been diagnosed with IBS… It’s like I’m on the outside looking in. I feel like, if I was diagnosed with Ulcerative Colitis… I could have found more people to talk to… I don’t think it would be as isolating as this (Charlotte, 25)

For Josie, her acceptance of living with pain has been acknowledged, however it is evident that she has pushed the illness identity behind her. Refusing to regard her Fibromyalgia as a CPI has allowed her to manage life with her symptoms. Whether this entails an acceptance or a way of concealing her symptoms from others is questionable:

I don’t talk about Fibromyalgia… I live with it… I try not to class it as a disability or even mention it… I just say, ‘I’ve got pain’… it doesn’t become my topic of conversation (Josie, 39)

Despite the growth in understanding of Arthritis, Amelia’s transcript depicted a sense of feeling frozen in time. Sharing her diagnosis would confirm her illness identity, bringing a sense of reality to her experiences, leading to a confrontation between being so young and yet experiencing the debilitating symptoms of her CPI:

It’s funny, ‘cos I find myself… I don’t tell anyone about it… Not even my closest friends know… If you tell anyone about it, it makes it more real… and we don’t really, like, have to be that person, with that tag on them (Amelia, 20)

#### Subtheme 3: Striving for normality

Holding onto a “sense of normality” following the vicissitudes a CPI can bring is critical for establishing coping strategies. However, the cyclic process of life with a CPI can bring troubling phases, navigating and adjusting to new demands, as they progress through the illness trajectory. Many face the challenges and adapt their illness perceptions and coping strategies accordingly, however, others struggle with each demand the illness brings. Sophie recalls the time of her diagnosis and her struggles around accepting her CPI, a battle which continues today:

I was in denial… it’s gonna pass somehow… I couldn’t accept it… Somehow, I still can’t accept it… there’s nothing good about having a condition… I just feel I am not complete (Sophie, 44)

Charlotte feels she cannot cope any longer with her illness, and rather than accepting her current situation, she steadfastly clings to the hope of future surgery. Whilst acknowledging the functional impairments she will face with a colostomy bag, her resounding faith in her surgery enables her to hold on to future perceptions that one day, her EWB will be restored, and life will be easier. Somewhat wistful in her perceived future, she affirms:

I can do day-to-day things… Get up… have some breakfast, go to work… I know that it’ll be over one day… which is really positive… I know that with stomas, it isn’t going to be easy… I know there are still complications… But it’s going to be so much better (Charlotte, 25)

For some, acceptance of their illnesses has enabled them to adopt a more practical approach to regain a more dynamic sense of normality to accommodate the cycle of the illness experience. For Amelia, however, her Arthritis has resorted in her concealing her symptoms and lifestyle restrictions from everyone, to establish and hold on to an outward sense of normality. By concealing her illness, she perceives she regains a sense of control over certain elements of her life, potentially due to the lack of control she has had with her illness in the past, or with her symptoms today:

I’m determined not to ever let it get in the way… So, I don’t (tell anyone). I want to be offered to go out in the cold… to build a snowman… decide whether I can do or can’t do it (Amelia, 20)

For patients with chronic pain, it could be that certain levels are manageable as their pain threshold increases. Anne, who was diagnosed several months earlier, draws on her own strength and determination to maintain a sense of normality. However, the short illness duration may have left her with little time to establish when she needs to reach out for help. This conflict between her lived experience and her perceptions of asking for help perfectly illustrate the difficulties many individuals face when adjusting to life with a CPI:

I’ve been in my bed sobbing… when your whole body feels like it’s completely shutting down on you, it’s fecking terrifying… if I called them, they’d come… I’m just like, this doesn’t count as an emergency (Anne, 19)

The process of adjusting to life was highlighted throughout Anne’s transcript. From attempting to see the positive side of finally receiving a diagnosis, to the reality of living with a heavily life impacting CPI, she is still learning about the nature of her illness identity, and how, at times, she slips back to her pre-illness self:

I just went out for my friend’s birthday… we sat on the ground for hours… it was icing my joints… I got home and the alcohol started wearing off… so suddenly I’m in a whole lot of pain all at once… so that sucked (Anne, 19)

For Jessica, who was diagnosed with Epilepsy as a child, her seizures have become an annoyance, regularly getting in the way as she strives for normality. The significant difference for Jessica, in comparison to the other participants, is that her seizures started due to medical malpractice, as opposed to developing naturally. Subsequently, her anger and distrust of HCP is clear:

I just wish… it would all just go away. Piss off and leave me alone… I’m okay… I’m just pissed off really. I do try to think positively, but it was their fault. Their error, and I’ve got to deal (Jessica, 24)

### Superordinate theme 2: A restrained reality

#### Subtheme 1: Fear of injustice

Chronic illnesses take many shapes and forms, with both clinically visible and invisible symptoms. Each of the participants within the current study were able to conceal their illnesses to an extent, however despite being able to avoid disclosing their illnesses to others, the majority still struggle with the impacts their CPI cause. Whilst having a diagnosis can lead to validity, providing a justification for the pain and suffering experienced, the women, whether with high or low prestige CPI, struggled with the anticipation of stigma. A distrust of others and a fear of being stereotyped has created a sense of unease surrounding their illness, and their place in society. Sophie explains how these fears evoke a need to justify her illness:

People assume it’s Diabetes type 2… So, I try to tell them that the type I’ve got has nothing to do with my lifestyle… It’s just not my fault… I was trying to explain to everyone, which is silly… I owe them nothing, no explanation (Sophie, 44)

Sophie’s concerns of what other people think of her was peppered throughout her transcript and has caused her to withdraw from society, fearing she may be judged for her illness being her fault. Conversely, for Jessica, her CPI is blamed on HCP, despite the lack of confirmation of malpractice. At 15, following being prescribed with an anti-epileptic, she developed Steven-Johnson Syndrome, causing 3rd degree burns all over her body. Through her lived experiences, she has become distrustful of HCP, and wary of all medications for fear of the repercussions, as she recalls:

People (doctors) say to me, like… Oh well this is a one in a million chance you’ll get this… and I say to them, yeah, well it might be one in a million chance, but it was one in a million chance I’d get Steven-Johnson’s Syndrome… and I got it (Jessica, 24)

For those with lower prestige illnesses, a hesitancy or inability to explain their CPI triggers perceptions of fear leading to withdrawal, indirectly negatively impacting their EWB by choosing a solitary lifestyle. The premise of the biopsychosocial approach recognises that each component interacts to impact health and well-being. Hence by self-isolating during symptom flares, Amelia may face further challenges in the future that could have a detrimental impact on her EWB and health-related quality of life, particularly with her future joint replacement. Amelia explains how she copes during difficult times:

I just say I’m working… or busy… It’s easier to be like, I’ve already made plans, when in fact I’m sat at home with a hot chocolate (Amelia, 20)

Fearful perceptions of how others see them can often be exacerbated at times, and for many, symptom flares can heighten self-consciousness, negative affect, and shame. Charlotte struggled painfully during her school years, due to her embarrassment around symptoms. Feeling unable to disclose her CPI to her friends left her feeling an outcast, impacting her psychosocial well-being and leaving her isolated for most of her adolescence, as she reflects:

… when I was a teenager… I couldn’t talk to anyone about it because it was so embarrassing, you know? I could talk to my parents, which was a godsend… but I didn’t have, when I was a teenager, I didn’t have anyone (Charlotte, 25)

Josie spent time reflecting on feeling neglected and dismissed, fearing that people believe she is exaggerating or making her symptoms up. Being unable to articulate or localise her pain and symptoms creates a need to justify herself and her illness, which has become an exhausting cycle, leaving her perceiving the negative responses of those around her:

You talk about the pain, which is not visible… People just undermine you… Oh maybe she doesn’t want to do something… Or, oh she doesn’t want to join us… you think, I would love to do that but I’m not physically and mentally able to do it… I just can’t do it (Josie, 39)

#### Subtheme 2: Bound by society

Careers and employment were highlighted by most within the narrative, through drastic changes in career direction or perceptions that their CPI made them unemployable, with their inability to find work evoking feelings of frustration and resignation. Sophie, with Diabetes, reflects of the sheer devastation of losing her job. Despite knowing that her inability to continue in the role was due to insurance matters, she continues to attribute the blame onto herself, truly believing that it is a failure of her abilities as an employee, as she reflects:

When they found out I had Diabetes… They sent me back home… But for me it was just worse than anybody else… As I believed I couldn’t work there because I have Diabetes, because I’m not a good worker (Sophie, 44)

Losing her place of employment soon after diagnosis of her CPI triggered a decline in Sophie’s self-esteem, causing her to question her abilities. With this comes an enforced change in identity and a challenge to an established sense of self. Charlotte’s inability to hold onto employment evokes a sense of disconnection, perceiving that as she does not contribute to society via work, she does not hold a place within the “adult world.” These beliefs were peppered throughout her transcript, as she feels that her illness is restricting her physically and emotionally to the extent that she is not living, but rather, just existing:

It’s sad not being able to work… It feels really… I don’t know, I just feel really useless… like I’m a spare part… I feel like I’m… a bit of a sponge (Charlotte, 25)

Jessica’s frustration implies her belief that she is unemployable due to her illness, potentially manifesting in a bitterness she feels towards the world, whereas there may be a level of unconscious bias towards her CPI on behalf of the employers and companies she has applied to:

I’ve applied for jobs that I’ve not been successful with… Erm… People with disabilities… I do get turned away… Due to me having Epilepsy… Due to being classed as disabled (Jessica, 24)

For Anne and Amelia, the onset of their illnesses disrupted their dreams, interrupting the career plans they had been building throughout their adolescent years. The restrictions placed on their lives simultaneously reduced their career options, leading them both quickly finding alternatives which may lead them to reflect on what could have been in the future. Anne’s transcript was peppered with humour, seemingly disguising her true feelings. For one so passionate about being on the stage, with her love of musical theatre evident throughout, the undertone of loss became apparent:

I used to be a dancer… I was going to do musical theatre as a profession. Things changed with that… immediately had to change my entire career path… it’s fine (laugh) (Anne, 19)

Amelia completed her training as a chef, identified as her true passion early on in her transcript, however this has been demoted to a hobby she uses to distract herself from her pain. Her eagerness to emphasize she is still employed provides an identity behind which to hide her CPI, using disguised coping strategies to meet the expectations of her employers and continuing to conceal the restrictions her symptoms place on her:

I’m currently not working as a chef. But I am working… I work with victims of crime… with the police… Then at the weekends, sometimes I work events as well… that’s often in summer, I don’t work well outside in winter… But I’ve got to work in winter… thermal-ing up, water-bottling it up (Amelia, 20)

#### Subtheme 3: A glimmer of hope

Positive affect is conducive to well-being and beneficial for managing the impacts of stressors within the illness trajectory, and the women used varying methods to uplift their EWB. Josie has adopted adaptive coping strategies such as mindfulness-based practices, including meditation, focusing on the food she is putting into her body, and going for walks outdoors. Living with her illness for the longest timeframe, it has taken time for her to understand the impact of her lifestyle on her symptoms, and understanding the mental-physical interplay:

It’s only now that it clocks on that my bad days are when I’m stressing… or when I’m working too much… erm… that’s when I suffer more… But if I’m happy and I’m out doing something that’s relaxing, that’s when I don’t have any pain (Josie, 39)

Feeling somewhat disconnected and separated from wider society, five out of the six participants found that spending time with their family and friends provided a perfect way to improve their moods, boost their EWB and provide a distraction from the challenges their illnesses bring:

Being around my family definitely helps and can change my mood… I’m really happy when I’m around my kids… I have nieces and nephews… and they take over my house and give me life (Josie, 39)

Talking of children giving her life provides Josie with temporary respite from the draining nature of her Fibromyalgia symptoms, fulfilling her with a sense of energy and happiness. Similarly, Sophie has found that being surrounded by her family is crucial for maintaining her well-being, and promoting positive affect, with time spent with her child of utmost importance. She speaks of her promise made to her daughter when she was born:

It doesn’t matter what kind of day I have… It is funny that you can cheat your brain by smiling… I said to myself that every day, I have to laugh and smile… whatever happens, at least 5 minutes… we have to do it (Sophie, 44)

Embodiment here is a key strategy. The physiological response of smiling is linked to a positive emotion for Sophie and her child, providing an uplift of her EWB, even if at times difficult to initiate. For Charlotte, her relationship with her mum has been extremely conducive to improve her EWB over the years, finding that just by sitting in her company takes her away from the isolation her illness regularly brings. As she explains:

I’ll just sit with my mum in the evenings… We don’t have to be actively watching something together… in the evenings, when I go down for dinner, I just hang around and talk to her a bit and it’s nice” (Charlotte, 25)

Anne perceives herself to be extremely fortunate for the fact that, despite the rarity of her CPIs, she has several friends who have either of her two illnesses. Having this friendship circle with each of her friends knowing and, critically, understanding the restrictions and implications of her illnesses, allows her to establish meaningful connections, resulting in positive affect, as she highlights:

One of the friends that I’ve made here… She happens to come from a family who all have fibro, so she knows how to deal… So, she’s excellent in helping me manage (Anne, 19)

Jessica also turns to her friend for support; however, her needs appear to be contrasting. Knowing about her seizures yet seeing the person inside provides Jessica with a level of support she desperately holds onto. She illustrates how her closest friend does not treat her any differently, in a stark contrast to how she perceives the rest of society views her, allowing her to somewhat escape the burden of illness, and subsequently boost her EWB:

I spend a lot of time with her… She’s the only one I talk to really… she knows about them (seizures), but she doesn’t constantly talk about them… I get sick of talking about it (Jessica, 24)

Conversely, Amelia steps back into her lifestyle of solitude to boost her EWB. Yet the activities she chooses offer mindfulness practices, providing a chance to escape the thoughts and experience of her arthritis, instead filling her with peace, as she illustrates:

I paint, or I will do some baking… I’ll make a point of doing something that I don’t normally have time for but love to do and will sit and do it (Amelia, 20)

## Discussion

The current research aimed to explore the subjective experiences of women living with a variety of high and low-prestige CPIs, the coping strategies adopted to manage their illnesses, and how they perceive their CPI influences their EWB. Jessica and Sophie have high-prestige researched and treatable illnesses, Charlotte and Amelia have diagnosable illnesses, perceived as middle to low-range, and Anne and Josie diagnosed with low-prestige CPIs (Album et al., 2017). Two key superordinate themes were developed, with three subthemes connected intersubjectively, evidencing the psychological struggles within the context of CPI, highlighting disparities experienced on meso (interpersonal), micro (societal) and macro (structural) levels. Adapting to their symptoms, whether with medical treatment or without, was challenging for all, as was regaining a sense of identity and control over their lives. Despite the variety of high and low-prestige illnesses, all participants struggled with establishing their place in society, and the fear of stigma, whether perceived, internalised, or experienced. Nevertheless, each participant had established methods of uplifting their EWB in times of struggle.

The down-prioritisation of low-prestige illnesses was evident within the narrative, with Amelia and Charlotte having to wait until their symptoms were highly impactive before treatment, and Anne and Josie left with no support. The delays in diagnosis, lack of available resources, and dismissal of reported symptoms illuminate the down-prioritisation of chronic pain conditions, despite the sheer number of people in the UK affected, demonstrating meso and macro level disparities (Sanchez, 2020). This left the women with a conflicted existential identity, perceiving that as they do not meet societal expectations of disabled, they do not fully belong to the disabled identity. This caused internal confliction in Anne and Charlotte and a denial of their illness experience in Amelia and Josie, which could likely have negative impacts on their mental health and EWB in the future.

Of all participants, only one was currently employed, and as Amelia was vocal about concealing her illness, she may have avoided the self-advocacy mechanisms necessary by many (Antao et al., 2013). Most talked freely of feeling detached from the real world, no longer contributing to society. They spoke of negative experiences with employers and an inability to continue in their desired roles due to their symptoms. Whilst indicative of discrimination of individuals with episodic illnesses, this could demonstrate inequalities of the gender construct on multi-dimensional levels, as reflected in the literature (Barr et al., 2023). Macro-level policies encourage equality and diversity within the workplace, with findings that with adequate support, working women with CPI report higher levels of well-being (Palstam et al., 2012). Yet the narrative was contradictory. Participants perceived their CPI made them unemployable, evoking feelings of frustration and a resignation to their chronically ill identity, resulting in an identity crisis. Perceiving their chronically ill identity suboptimal compared to the “gainfully employed,” a finding mirrored across the literature (Dickson et al., 2008).

Multidimensional discrimination was perceived across the narrative, potentially reflective of disparities within the gender construct (Barr et al., 2023). These disparities were likely magnified with most participants being adolescents at the onset of their symptoms, and despite evidence indicating an increased prevalence in early onset conditions, this remains an under-researched area (Costello et al., 2021). Most spoke of striving to gain a sense of control over their illnesses to increase their EWB, and the majority appeared to be aware of the interplay between CPI and EWB. A variety of coping strategies were adopted, and all participants turned to their support network to alleviate the stressors associated with their illness, to enhance their capacity for coping. In accord with prior research, the high levels of support highlighted by participants are typically associated with positive illness perceptions, increased adaptive coping strategies and self-management behaviours (Cao et al., 2017).

### Limitations and future research directions

Current research into the prestige hierarchy focuses on HCP perceptions which, despite the developing understanding of medicine, remain ingrained, and subsequently the discourse needs to change on macro levels for change to permeate through structures to benefit those experiencing inequities. Future research recommendations would be to systematically review societal perceptions of CPI prestige to provide a broader representation of the disparities in healthcare caused by such perceptions. A failure to address the perceptions of prestige will see a continued down-prioritization of these CPIs disproportionately affecting women and other marginalized groups within healthcare settings. The homogeneous group of women with various CPIs was ideal for IPA analysis, however, an exploration of the medical hierarchy through qualitative surveys to provide more breadth of cases would be beneficial. The term EWB could be a limitation, as participants’ perceptions may not reflect the full extent of the impacts of CPI. Providing a measure of EWB before the interviews may prompt participants to consider abstract ways their lives are impacted by their CPI, whilst providing a statistical understanding to the research. Additionally, using a university student platform for recruitment may have introduced motivational or selection bias. The justification was to ensure an appropriate sample had been recruited promptly due to time restraints, however, future use of social media or healthcare settings may be more appropriate.

### Conclusion

The current study highlighted commonalities and variations among women living with a variety of high and low-prestige CPIs and the ways their illness impacts their EWB. The variance in CPIs highlighted both high and low-prestige illnesses, typically accepted or ambiguous within society. Being diagnosed with any CPI, no matter its perceived position on the medical hierarchy can be devastating, as patients not only confront their diagnosis but must navigate a disjointed identity and sense of place in society throughout the illness trajectory. However, those with lower prestige illnesses evidently struggled with the concept of not quite meeting societal expectations of disabled, hence lived with a conflicted identity or concealed their symptoms entirely. The ranking of CPIs should not be reflected in the support and treatment available to patients, and subsequently, the historical perceptions must be addressed on a structural level so that the illness experience of all patients can be validated within healthcare settings and society. It is critical that the reductionist discourse surrounding the medical hierarchy is promptly modified to address economic inequities. In doing so, invalidation and potential subconscious bias within healthcare settings can be reduced, and more information and resources can be made available and accessible to patients.
